# Effect of a Narrative-Based Online Course Aimed at Reducing Stigma Toward Transgender Children and Adolescents: Longitudinal Observational Study

**DOI:** 10.2196/59605

**Published:** 2025-01-09

**Authors:** Merlin Greuel, Van Kinh Nguyen, Doron Amsalem, Maya Adam, Till Bärnighausen

**Affiliations:** 1 Heidelberg Institute of Global Health Heidelberg University Heidelberg Germany; 2 Department of Psychiatry Irving Medical Center Columbia University New York, NY United States; 3 Department of Pediatrics Stanford School of Medicine Stanford University Stanford, CA United States; 4 Center for Digital Health Stanford School of Medicine Stanford University Stanford, CA United States; 5 Department of Global Health and Population Harvard T H Chan School of Public Health Harvard University Cambridge, MA United States; 6 Africa Health Research Institute Somkhele South Africa

**Keywords:** stigma, transgender, children, adolescents, mental health, online course evaluation, entertainment-education, narratives

## Abstract

**Background:**

Stigma toward transgender children and adolescents negatively impacts their health and educational outcomes. Contact with members of stigmatized groups can dismantle stereotypes and reduce stigma by facilitating exposure to the unique cognitive and emotional perspectives of individuals within the group. Recent evidence suggests that video-based contact interventions can be as effective as face-to-face encounters, but challenges lie in protecting the identities of transgender youth, since many of them live in stealth.

**Objective:**

This study aims to evaluate the impact of an animated online course, rooted in authentic, personal narratives, on course participants’ stigma toward transgender youth.

**Methods:**

The online course was offered free of charge on Coursera and contained 19 teaching videos (3-7 minutes each), intermittent practice quizzes, and discussion prompts. Using real voice recordings of transgender children and their caregivers, the videos were designed to elicit empathy and transmit knowledge. All videos conveying the narratives of transgender youth were animated to protect their identities. A total of 447 course participants, distributed around the globe, completed pre- and postcourse surveys. While the course primarily targeted parents and caregivers of transgender youth, it was open to anyone with a Coursera account. The survey was based on the Transgender Attitudes and Beliefs Scale but modified to reflect the context of parents and caregivers. Using a 5-point Likert scale, it contained 5 questions that captured participants’ levels of transgender stigma. Results of the pre- and postcourse surveys were then compared.

**Results:**

The results were obtained in January 2023. Baseline levels of stigma were relatively low (18/25 across all questions, with 25 representing the lowest possible levels of stigma) and decreased further after completion of the course (to 19/25 across all questions, *P*<.001). A multivariate ordinal probit regression showed that, depending on the question, participants were 7%-34% more likely to endorse statements that indicated the lowest levels of stigma after completing the course. The course was equally effective across all demographics represented in our participant population.

**Conclusions:**

Our findings document a significant reduction in stigma toward transgender youth in participants who chose to enroll in the first animated, open online gender health course, rooted in the authentic narratives of transgender youth. Stigma levels decreased significantly after taking the course, even among participants whose baseline levels of stigma were low. Future interventions should include participants with more variable baseline levels of stigma, ideally in the setting of a randomized controlled trial. Despite its limitations, this evaluation adds to the existing evidence that digital, contact-based antistigma interventions, animated to protect the identity of the narrators, can effectively reduce stigma toward transgender youth.

## Introduction

Adolescence is a period marked by change, self-discovery, and significant vulnerability to mental health issues [1]. Children and young adults who realize that their gender identity differs from their sex assigned at birth are especially prone to poor mental health outcomes: transgender youth endure elevated rates of depression, self-harm, eating disorders, and suicidality compared with their nontransgender peers [2-4]. In addition, the educational outcomes of gender-diverse children often lag behind those of their cisgender peers [5]. Thus, the stressors that affect gender-diverse children and adolescents can have a profound effect on individuals’ academic performance and the course of their future lives.

Stigma toward transgender youth contributes to the observed differences in mental health. According to the gender minority stress theory, many of the health disparities observed between transgender and cisgender individuals can be attributed to stressors emerging from the stigma faced by transgender people, also called “transphobia” [6,7]. The chronic stress associated with transphobia results in poor mental health outcomes with repercussions even on physical health, as evidenced by elevated rates of diabetes, hypertension, and death by suicide [7]. This is especially true for individuals with multiple disadvantaged statuses such as low-income, transgender women of color [7,8]. Stigma can affect individuals’ health directly, for example, through chronic activation of the body’s stress response and increased cortisol output [7], or indirectly by decreasing access to the housing market, education, and employment [7,9], all critical social determinants of health.

Depending on the academic discipline, stigma has been studied from different angles, using diverse terminology [7,9]. Stigma may be defined as “a severe social disapproval due to believed or actual individual characteristics, beliefs or behaviors that are against norms, be they economic, political, cultural, or social” [10]. More recently, neuroscience has offered a fresh perspective on stigma, describing its cognitive, emotional, and behavioral dimensions [11-13]. Stigma can be considered a defensive reaction resulting from an innate fear of individuals who differ from the self and collectively form an “outgroup” [11]. Functional neuroimaging studies show that stigma [14,15] and perceived outgroup membership [16,17] can decrease the levels of empathy felt for others. As a consequence, members of the outgroup are depersonalized and viewed as more homogeneous than ingroup members, thus encouraging prejudice, negative stereotyping, and antisocial behaviors [18].

Contact with a member of a stigmatized community can dismantle stereotypes and facilitate entry of the previously stigmatized person into the in-group [19,20]. Intergroup contact is considered the most effective means of reducing stigma [20,21]. Experimental evidence suggests that video-based contact interventions may be as effective as face-to-face encounters while being less expensive and easier to disseminate [20,22-24]. The conceptual framework of Jankowski et al [20] for social contact-based video interventions highlights the importance of addressing the cognitive, emotional, and behavioral dimensions of stigma [20]. By targeting all of these components, viewers can empathize and identify with the protagonists, thereby reducing intergroup anxiety [20] and with it the perceived barriers between groups.

By allowing the public to experience the cognitive and emotional perspectives of transgender youth, authentic, personal narratives lend themselves to antistigma interventions [18]. Narratives can provide a nonthreatening context to establish vicarious social contact with others who might previously have been met with anxiety or mistrust. Narrative stories have been shown to elicit more compassion, favorable attitudes, and beneficial behavioral intentions toward stigmatized groups when compared with nonnarrative stories [25]. High capacity for engagement, as well as the compassionate and affective reactions evoked by the narrative format, are thought to trigger empathic attitudes in the audience [25] that may translate into nonstigmatizing behavior. Through empathy and knowledge transfer, transgender individuals may leave the “outgroup” and enter the “ingroup,” allowing their nontransgender peers to view and treat them as one of their own. Yet, few studies have explored the use of video-based narratives for reducing the stigma against transgender youth. These studies found that social contact–based videos have the potential to increase knowledge about transgender youth and to decrease transphobia [26,27]. However, care needs to be taken to protect the identities of transgender youth who share their stories, since many of them live in stealth [28]. To our knowledge, no study has examined the effect of integrating animated short videos, featuring authentic voices and narratives, into an online course aimed at reducing transphobia. Thus, this article contributes to the existing literature by integrating evidence-based methods to reduce transgender stigma into a novel, digitally scalable format that protects the identities of the narrators. The benefit of an online course over interventions with exposure to a single video is the possibility to expose participants to content over a longer time period, enabling a more in-depth discussion of course material, repetitions of core concepts, and assessments that stimulate subjects to actively engage with the topic of interest.

Here, we evaluate the effect of the online course “Health Across the Gender Spectrum,” rooted in entertainment narratives, on participants’ attitudes toward transgender youth. The aim of the course was to promote a gender-inclusive society by establishing virtual contact with the transgender community. The narratives were unique in that they included real voice recordings of transgender children as well as their parents and caregivers and they were animated to conceal the identities of the storytellers. The course was made freely available to the public, with some targeting toward parents and caregivers of children whose gender identity differs from their sex assigned at birth. This target group was chosen because adolescents’ “coming out” as transgender can significantly deteriorate family relations [2] due to a lack of acceptance of family members. Family rejection of transgender children and adolescents is strongly correlated with mental health issues, substance use, suicidality, and sexual risk [29-31]. On the other hand, family support of transgender youth protects against depression and improves their quality of life [32]. We analyzed pre- and postcourse survey data to determine whether participants’ attitudes toward transgender youth changed after completing the course. Our hypothesis was that subjects would show lower levels of stigma toward transgender youth upon completion of the course. This evaluation may help pave the way for other innovative, empirically verified interventions to reduce family and caregiver stigma toward transgender individuals, thus improving their health and life outcomes [29-32].

## Methods

### Evaluation Design and Approval

This online course evaluation was designed as a virtual contact-based exposure study with a pre-post design, using a convenience sample. The CHERRIES (Checklist for Reporting Results of Internet E-Surveys) [33] was used to guide this article.

### Online Course and Participants

The course “Health Across the Gender Spectrum,” still available free of charge on the Coursera online platform as of publication [34], was set up as a 3-week program (8 hours of instruction in total) consisting of 3 modules: “What is Gender Identity?” “What is the Gender Spectrum?” and “How Do We Create a Gender-Inclusive Society?” Coursera is a global massive open online course provider based in the United States and used by universities and similar organizations to offer online courses, certificate programs, and degree courses, some of which are free of charge while others require a tuition fee. Participants had no fixed time schedule and could decide the pace of their progress on their own. The language of instruction was English. Conceived by the Stanford School of Medicine under the leadership of our coauthor (MA), learners were introduced to the experiences of 6 transgender children and their families. Through illustrated stories and 19 short teaching videos, participants explored the topics of gender identity and the gender spectrum. Stanford physicians, K-12 educators, and transgender faculty members offered practical advice for parents, health care providers, teachers, and others interested in exploring gender identity in children and adolescents.

Apart from a Coursera account, there were no prerequisites for enrollment. That is, while the course was geared toward parents and caregivers of transgender and gender-diverse children and adolescents, anyone was eligible to enroll. The course was announced to learners enrolled in the Stanford Child Nutrition and Cooking Course, also available on Coursera. Coursera also independently announces new courses to registered account holders. Each of the 3 modules was divided into subsections that sought to answer common questions such as “What’s the difference between gender identity and sexual orientation?” The answers were then provided by a series of short, animated educational videos, interspersed with the narrative videos. Each subsection contained one practice quiz with one or two true-false questions, which required a response to move on to the next video. Before advancing to the next subsection, learners had the option to answer a voluntary discussion prompt. At the end of the last section of each module, participants were asked to complete a graded quiz of 4-6 questions. A score of 80% or higher was required to move on. Learners could repeat the quiz as often as they wished. An overview of all modules, sections, and titles of the videos and discussion prompts can be found in Multimedia Appendix 1.

We did not store any participant information other than the survey responses submitted by the learners. We had access to anonymized data from Coursera, such as the IP addresses of those who submitted surveys. These data did not personally identify any participants. Subjects were informed about the purpose of the surveys and their approximate length (3 minutes).

### Video Animations

**Figure 1:**
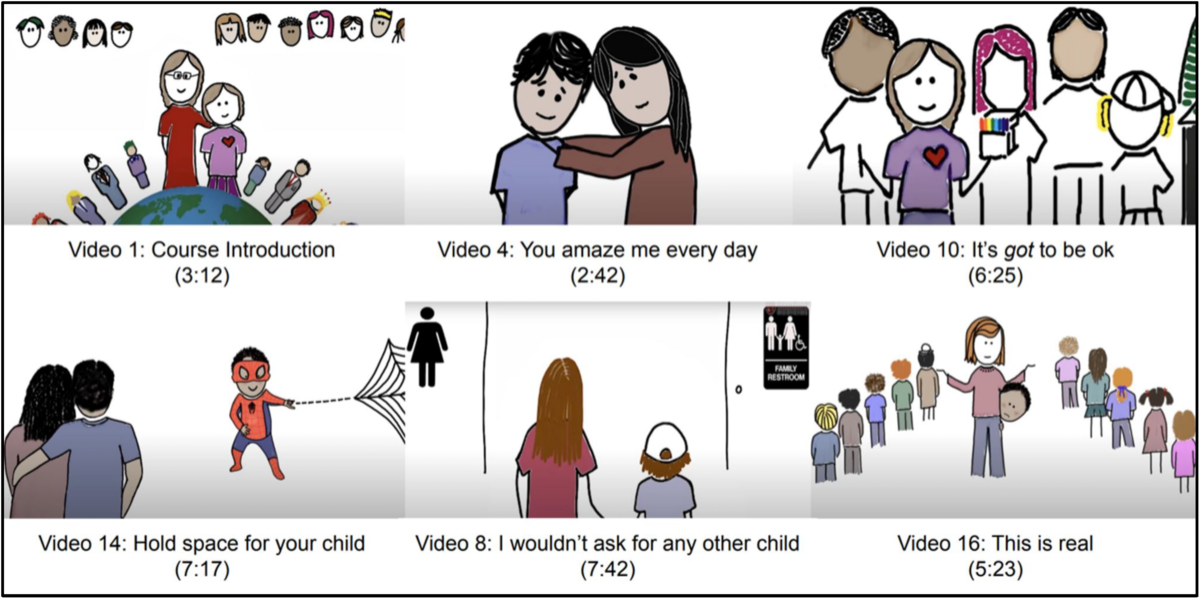
Sample scenes from narrative course videos with titles and run-times.

A total of 19 teaching videos (3-7 minutes each) were developed by our coauthor (MA) at the Stanford School of Medicine, with input from faculty at the Pediatric and Adolescent Gender Clinic. The videos featured universally accessible animations, appropriate for all cultural, ethnic, and racial contexts. This raised the likelihood that viewers could identify with the protagonists no matter their own cultural identity and origin. In some of the videos, MA interviewed faculty experts from the Stanford Pediatric Gender Clinic as well as 2 transgender faculty members, who appeared in person. The transgender faculty members shared personal anecdotes and experiences relevant to the course theme. However, depending on the 3 modules of the course, the animated content predominated and ranged between 75% and 100%. The narrative videos’ hallmark was their story-telling approach, using the real voices of transgender children, parents, and caregivers to make the animations come alive. Rather than being didactic, these videos were designed to establish vicarious social contact between the viewers and transgender children as well as their families and caregivers, in a nonthreatening context. The use of animation effectively protected the identity of the young people who shared their stories and allowed us to visually emphasize the importance of diversity and inclusion in all vignettes. The emotional appeal of the video narratives relied heavily on the characters’ descriptions of their feelings, which were mirrored in the animations and underscored by customized soundtracks. The aim was to enable viewers to experience the cognitive and emotional perspectives of the protagonists, thereby triggering compassionate and affective reactions and empathy. Some of the videos focused more on individuals’ personal experiences, while others transmitted knowledge about gender identity and related topics. The language was simple and avoided medical jargon to reach viewers with diverse educational backgrounds. Figure 1 shows select scenes from the narrative videos. A complete list of course videos can be found in Multimedia Appendix 2.

### Survey

After enrollment, participants received an email in which they were asked to complete an open precourse survey. The survey was hosted by Stanford School of Medicine’s Qualtrics account and appeared on a single page. Demographic information was collected on the second page. Qualtrics is a platform for online experiments and data collection [35]. To match pre- and postcourse surveys, we used Coursera’s user ID hashtags. In addition, participants were asked to generate a unique survey ID as a backup. We also used IP addresses, locations, demographic information, and dates of submission to identify any falsely matched surveys or multiple submissions. The questionnaire was informed by the Transgender Attitudes and Beliefs Scale (TABS) [36]. We chose to abbreviate and modify the TABS for the context of parents and caregivers because the original TABS contains 29 items, which we deemed too long for a survey taken on a voluntary basis, with no financial incentives or compensation. Our questionnaire contained 5 items aimed at measuring preexisting stigma against transgender children and adolescents. The statements were based on the first 2 subscales of the TABS, which explore interpersonal comfort with transgender individuals and sex or gender beliefs. We modified the items to better reflect the perspective of parents and caregivers of transgender children and adolescents. For example, one of the items in the original TABS reads “All adults should identify as either male or female” (Q3.13, subscale 2) [36]. We changed this item to “It is important that all children learn to identify themselves as either a boy or a girl” (item 5 in our questionnaire). Participants responded through a 5-point Likert scale and an additional not applicable option. The answer choices ranged from “strongly disagree” to “strongly agree.” Please refer to Multimedia Appendix 3 for the complete questionnaire, and to Multimedia Appendix 4 for an item-by-item overview of how the original TABS items were modified. The technical functionality of the survey was tested before its launch. The data were collected between 2017 and 2023.

After completing the course, learners received another email asking them to fill out the same questionnaire, thus allowing for pre-post comparisons. In both surveys, participants were instructed to generate a unique, anonymous identifier to match the surveys in the analysis.

### Data Analysis

Whenever possible, the pre- and postcourse surveys were matched for each participant, creating a fully matched sample of individuals. We used a multivariate ordinal probit regression for matched data, in which the 5 questions were modeled jointly [37]. This approach treats the Likert response as an ordinal scale providing a better theoretical interpretation and statistical inferences (compared with a nonparametric or normally distributed response assumption, eg, in a change point analysis) [38]; furthermore, potential correlations between the questions are taken into account [39]. Matched individuals were modeled with a random intercept to allow for individual heterogeneity (Multimedia Appendix 5). An ordinal probit regression can model the relationship between one ordinal-dependent variable (in our case, the answer choices of the Likert scale) and one or more independent variables [40]. A multivariate ordinal probit model also takes into account the potential correlation among the responses across the 5 questions [37]. First, we defined that an answer was in the “positive” direction when it reflected low or very low levels of transgender stigma. For the first 3 questions, a “positive” answer meant that participants agreed (score of 4) or strongly agreed (score of 5) with the respective statements; for the last 2 survey items, a response in the “positive” direction was reflected by respondents’ disagreeing (score of 4) or strongly disagreeing (score of 5) with the statements. We then calculated the cumulative probabilities for each Likert category in the pre- and postcourse surveys; in other words, we determined the likelihood that a subject chose a specific answer (Likert category) for each question. Finally, we calculated the ratio between the probabilities for each question-and-answer choice (Likert category) in the post- versus precourse survey. This ratio describes the relative effect of the intervention. For instance, a relative effect (ratio) of “1” indicates that respondents’ likelihood of choosing a respective answer choice after completing the course was just as high as before the course. A relative effect of “0.5” means that participants were half as likely to choose a respective answer after completing the course, compared with the precourse condition. Conversely, a relative effect of “2” indicates that subjects were twice as likely to select a respective answer choice after the course.

Since respondents often included erroneous identifiers, not all surveys could be matched. Thus, as a robustness check, we performed a secondary analysis on a propensity score 1:1 matched sample [41] (Multimedia Appendix 6). We used a probit regression as described above. The matching procedure used nearest neighbor matching and sampling with replacement [42].

### Ethical Considerations

We obtained ethics approval from Stanford University’s institutional review board (protocol #76972). Completion of the survey was optional and not a prerequisite for beginning the course, and no incentives were offered to participants. The data collected were anonymized and did not allow for the personal identification of subjects.

## Results

### Demographics

**Figure 2:**
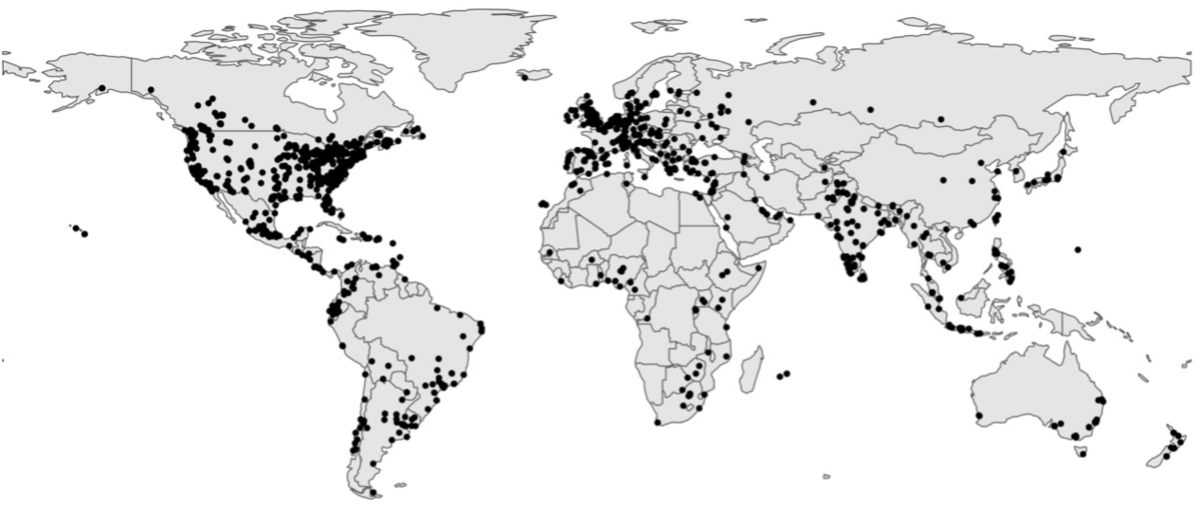
Geographic distribution of the sample, showing both pre- and postcourse participants, including those that could not be matched regarding their pre- and postcourse surveys.

By January 2023, 3878 learners completed the course. Of these, we obtained 1591 survey records. A total of 447 participants (12% of all 3878 learners) could be fully matched based on subjects’ unique survey IDs. Coursera’s user-ID hashtags were not used due to a technical issue on the part of the platform. We also used data such as IP addresses, location, submission dates, and demographic information to identify potential mismatches or multiple submissions between pre- and postcourse surveys. If we found any mismatch or multiple submissions, we did not include the submission. Of the 447 learners, 377 (84%) were female, 406 (91%) had college-level education, and 283 (63%) identified as White. A total of 229 (51%) of participants completed the survey in 2017. A total of 200 participants (45%) completed both, the precourse and the postcourse survey within one day. Table 1 shows an overview of the subjects’ social demographics for the matched sample. Figure 2 depicts the geographic distribution of all recorded participants, containing both pre- and postsurvey data.

**Table 1:** Descriptive social demographics and responses of the fully matched sample.

Characteristics		Values
Age (years), mean (SD)		37.29 (14.85)
**Sex, n (%)**		
	Female	377 (0.84)
	Male	47 (0.11)
	Gender-diverse	23 (0.05)
**Education, n (%)**		
	College	406 (0.91)
	High school	41 (0.09)
**Race, n (%)**		
	Asian	45 (0.10)
	Black	14 (0.03)
	Latino	79 (0.18)
	Mixed or other	26 (0.06)
	White	283 (0.63)
**US-based, n (%)**		
	No	280 (0.63)
	Yes	167 (0.37)
**Year of survey completion, n (%)**		
	2017	229 (0.51)
	2018	55 (0.12)
	2019	17 (0.04)
	2020	70 (0.16)
	2021	37 (0.08)
	2022	28 (0.06)
	2023	11 (0.02)
**Time between pre- and postsurveys, n (%)**		
	Day	200 (0.45)
	Week	66 (0.15)
	Month	129 (0.29)
	Month or more	52 (0.12)
**Total score, median (IQR)^a^**		
	Precourse	18 (14-20)
	Postcourse	19 (16-20)

^a^Wilcoxon signed rank *P* value <.001.

Of the participants in the fully matched sample, 167 (36%) were located in the United States, the country most highly represented in the course. The US-based subjects were distributed across the country, following a pattern that roughly reflects the country’s population density (Figure 2). However, 280 (63%) learners were located outside of the United States, distributed across all continents with larger clusters in Europe and Latin America.

### Levels of Stigma

The 5 questions had a Cronbach α of 0.83, reflecting the questionnaire’s high level of internal consistency. Given that each of the 5 questions allowed for a maximum possible stigma score of 5 (and a minimum of 1), the maximum possible score for all 5 questions combined (indicating the lowest possible levels of stigma) was 25. The median of the total score across all 5 questions before the course was 18 (IQR 14-20). This median increased significantly to 19 (IQR 16-20) after the course (Wilcoxon signed rank test, *P*<.001).

**Figure 3:**
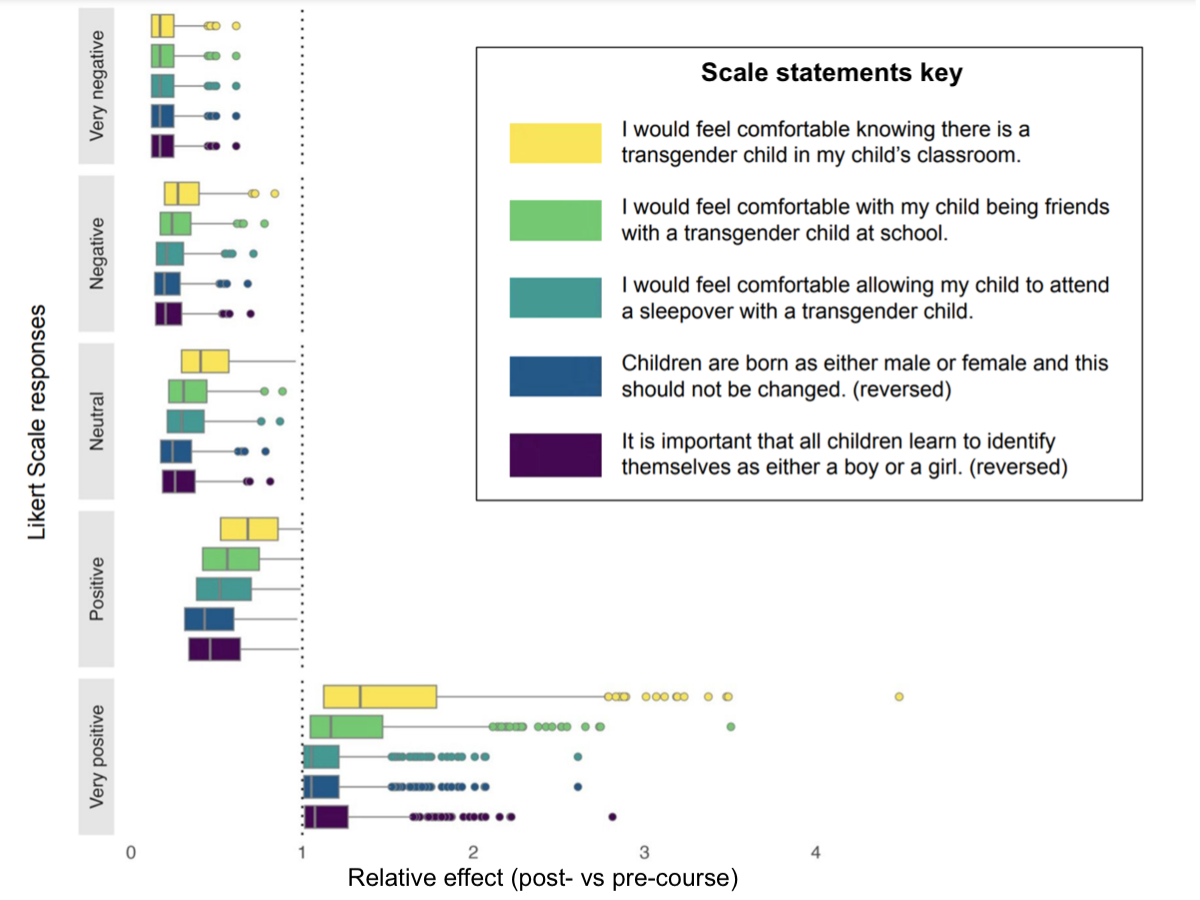
Marginal effect of the course on the responses to the 5 questions. The relative effect was computed as the ratio between the postcourse and the precourse predicted probabilities for each survey item.

Figure 3 shows the effect of the course on the fully matched data, showing separate results for each of the survey items. After the course, participants had a higher likelihood of answering very positively (strong agreement with the first 3 statements, strong disagreement with the remaining 2 items). At the same time, they were also less likely to provide negative, neutral, or less positive answers. The effect of the course varied largely between individual participants. While acknowledging that our sample was quite homogeneous in terms of demographic variables (refer to Table 1 and Discussion below), we found no significant differences in efficacy across the demographics represented in our participant population. The results of the secondary analysis on a propensity score 1:1 matched sample were consistent with those presented above (Multimedia Appendix 6).

## Discussion

### Principal Findings

In this survey-based evaluation of the social contact–based online course Health Across the Gender Spectrum, we observed a significant decrease in participants’ stigma toward transgender children and adolescents. Baseline levels of stigma were low but decreased further upon completion of the course. This effect was observed for all survey items and across all demographic groups. Despite its limitations, the findings of this evaluation provide the first documented evidence that an online course rooted in animated narratives may effectively reduce participants’ stigma toward transgender youth. In addition, the fact that the online course reached subjects across all levels of education, gender, age, ethnicity, or location is another important revelation of this study with the potential to guide future interventions.

The course significantly reduced stigma toward transgender children and adolescents across all question items, despite low baseline stigma levels. After completion of the course, participants were more likely to respond “very positively” and less likely to choose answers in the other categories. This means that there was a general shift in responses toward the “very positive” category, which reflected the lowest levels of stigma. This effect was observed for all questions but especially pronounced for the statements regarding the innateness of gender and the importance that children learn to identify as a boy or a girl. These 2 statements were arguably the most complex to understand for a learner who had not been exposed to the course. Moreover, they represented the highest level of escalation together with the question regarding the acceptability of sleepovers with transgender children, meaning that they had been designed to maximize the discomfort of participants with high levels of stigma. The fact that the strongest change was observed for these statements implies that subjects with higher preexisting levels of stigma or less informed participants may have benefited the most from the course.

Not surprisingly, learners who enrolled in the course showed relatively low levels of transgender stigma even before completing the intervention. This makes sense because the course likely attracted learners with socially progressive attitudes and an openness to learn more about transgender individuals. This is a limitation of this study, as individuals with higher levels of transphobia are presumably less likely to enroll in this course. Nonetheless, the fact that we were able to document a measurable reduction in stigma, even among this enlightened group, suggests that the stigma-reducing effect of the course could be even greater in populations that are less accepting of gender diversity in children. Future studies, ideally in the form of randomized controlled trials, may explore the stigma-reducing effect of this course or similar content within a more heterogeneous sample that is representative of the population as a whole.

The findings of our evaluation confirm previous research that demonstrated the stigma-reducing effect of social contact–based antistigma interventions [26,27]. This article adds to the literature by demonstrating that an online course with real learners (rather than participants recruited in a controlled experimental setting) achieved similar results. Furthermore, this is the first open online gender health course to experiment with the use of animation to protect the identities of transgender youth sharing authentic stories. Research in the field of neuroscience may help to explain why the course was effective. Stigma is thought to encompass a cognitive, emotional, and behavioral dimension [11-13]. The power of social contact–based video interventions is that they can address all of these components [20]. Emotion is a core feature of stigma, which often manifests as reflexive disgust, fear, and disapproval [11]. The brain regions responsible for emotional responses, the amygdala and insula, generally have a shorter latency to respond to aversive stimuli (eg, those inducing disgust) than cortical regions [11,43,44]. Thus, with the emotional response preceding cognitive modulation, emotions play a primary role in shaping individuals’ proneness to exert stigmatizing behavior.

Direct contact with a member of a stigmatized community can reduce the negative emotions associated with stigma [20]. However, virtual contact may also be effective, as exemplified by an intervention that established intergroup contact with individuals enduring schizophrenia using an electronic chat [20,45]. Participants who had been in virtual contact with a schizophrenia patient exhibited lower levels of anger, fear, and stereotyping compared with those who had been in virtual contact with a person who did not suffer from a mental illness [45]. In fact, even just imagining a positive encounter with a schizophrenia patient can reduce anxiety and negative stereotypes [46], demonstrating the power of social contact—be it direct, virtual, or imagined—in shaping our emotions. In a similar way, the video-based virtual contact in our online course might have alleviated the negative emotions associated with transgender stigma, thereby contributing to the reduction in stigma scores observed in the postsurvey.

While emotions are key, stigma is more than just an affective response. It also includes cognition. Implicit stigma describes attitudes and biases that are outside of conscious control [47,48]. It is impulsive and depends on associative links [11]. In contrast, explicit stigma occurs within conscious awareness and is self-reportable and regulatable through cognitive processes [11,48]. Explicit stigma relies on factual knowledge and reflection [11]. Thus, an effective contact-based online course should also transmit knowledge. Contact-based interventions have proven effective when they portray characters that deviate from negative stereotypes in moderate ways; that is, by focusing on the characters’ struggles and stories of hope instead of their accomplishments [20]. This is precisely what our videos do. In the video segments, animated children (narrated by real transgender youth) describe their journeys—their initial doubts about their gender identities, their feelings of shame and guilt, their fear of “coming out” and all of its consequences. Interviews with 2 transgender Stanford faculty role models give caregivers and parents hope by sharing how they successfully navigate the challenges of being transgender in academia. Finally, short clips embedded between the video sequences of transgender experiences also transmit facts about the concepts of gender identity, sexual orientation, the gender spectrum, the medical classification of transgender, gender-affirming management options, and the creation of a gender-inclusive society. A lack of subject knowledge supports the formation of stigma [49], so the aim of these supplementary videos was to provide facts that were easily comprehensible to a global audience. Thus, the effect of our video-based online course may have been underscored by the fact that its contact-based approach addressed both the emotion and cognition underlying stigma. The interaction between emotion and cognition gives rise to the concept of empathy—the ability to be sensitive to, comprehend, and vicariously experience the thoughts and feelings of others.

Fear of individuals who differ from the self are categorized internally into an “outgroup” [11]. Those who exert stigmatizing attitudes view “them” as decisively different from “us” [50,51]. This assumption prevents individuals from identifying with others: people use their self-knowledge, mental states, and experiences to infer what others might think or feel [50]; that is, to engage in the theory of mind [52]. If others are deemed fundamentally different, self-referential thinking is reduced [50], and with it, empathy. A neurobiological correlate of stigma is a reduction in the activity of the medial prefrontal cortex [52], a part of the brain that is thought to modulate empathy as it is involved in judgments about others and in viewing others as “human” [52]. As a consequence, members of the outgroup are depersonalized and viewed as more homogeneous than ingroup members, thus encouraging prejudice, negative stereotyping, and antisocial behaviors [18].

Since neural projections connect the amygdala and insula (responsible for emotion) to the ventral medial prefrontal cortex (modulating empathy), it is likely that affective responses are integrated into the theory of mind and empathy processes [52]. Moreover, emotions and empathy influence stigma-related cognition and behavior (and vice versa) [52,53] through a complex interplay involving other areas of the brain: cortical structures such as the temporal lobes and the inferior frontal gyrus encode and store stereotype concepts as semantic or social object (person) memory, which can be selected and transferred into working memory to influence judgments and behaviors [52]. The anterior temporal lobe, particularly involved in the representation of social knowledge (attributes describing people), is densely interconnected with the medial prefrontal cortex (the neural center for empathy) and the amygdala (implicated in emotion) [52,54]. Thus, it becomes evident that the various subcomponents of stigma—implicit versus explicit, with emotional, cognitive, and behavioral elements—can be traced to neurobiological correlates that work in a concerted action, determining individuals’ capacity for empathy.

The videos in our course were designed to trigger a strong empathic response in viewers, containing emotionally salient stimuli such as the use of transgender children’s original voices that explained their experiences, frustrations, and feelings of distress. Compelling visuals and soundtracks were intended to further support an empathic response in viewers. At the same time, the knowledge transmitted throughout the course provided participants with the necessary context to understand the medical and social background of transgender adolescents. By acknowledging the multidimensional nature of stigma, the course notably decreased participants’ stigma against transgender children and hopefully contributed to reducing the third component of stigma—the behavioral (enacted) one. Future studies designed as more rigorous randomized controlled trials may build on this article and include a behavioral endpoint. Furthermore, the emotional and empathic response of viewers may be tested in laboratory-based fMRI studies or by using online platforms that track viewers’ facial reactions through a webcam. The former may elucidate the real-time impact of the intervention on the brain regions involved in stigma in a limited number of individuals, while the latter may be easier to implement in large-scale interventions with a greater sample size.

### Limitations

This evaluation has several limitations. First of all, participants were matched ad hoc, resulting in a large number of subjects that could not be matched. Thus, the size of the matched sample represented only a third of the overall number of records. We partly alleviated this issue by performing a secondary analysis using propensity score matching to include more participants. This analysis supported our results. Second, our evaluation lacked a control group. Future studies should include a control group that is not exposed to the course. In addition, the time period between the pre- and postcourse surveys was variable among participants. It is unclear how this may have affected subjects’ levels of stigma, although variable completion rates likely reflect the reality of most online courses. Another limitation was that participants enrolled voluntarily in the course. Learners who enrolled in the course likely shared characteristics that differ from the population as a whole, such as holding more socially progressive attitudes or an elevated interest in transgender issues. This is reflected by the fact that our sample was largely female, college-educated, and White. Those who enrolled had low baseline levels of stigma, and the course was likely unappealing to individuals with high levels of transphobia. Future studies should ensure random sampling and the inclusion of participants with diverse attitudes toward the transgender community and more balanced demographic features. Furthermore, as described above, stigma is inherently complex, and the questionnaire used is a simplistic method that might not capture the full dimension of stigma. The questionnaire had not been validated before this evaluation. We found no validated surveys that were appropriate for measuring transphobia toward youth within the parent-child context of the course content. To the best of our knowledge, there are no widely used, validated surveys measuring transphobia toward children and adolescents within this context. Another limitation of this intervention is that it remains unclear how much of the course’s effect may be attributed to the narrated, animated content versus the appearance of guest speakers, discussion prompts, or quizzes. Yet, given that the majority of the content was animated, it is likely that the narrated, animated format of the course was the main active ingredient in the outcomes observed here. Finally, our intervention lacked any follow-up assessment. Future studies should include follow-up evaluations to explore if subjects’ improvements in transgender stigma are stable over time.

### Conclusion

This evaluation of transphobia in online learners enrolled in an animated, story-based course adds to the limited existing evidence that digital, contact-based antistigma interventions can be as effective as face-to-face approaches. Our findings make a case for readily scalable, digital contact-based interventions that use animation to protect the identities of individuals sharing their lived experiences. Ongoing advances in the neuroscience of stigma will further increase our knowledge of how animated educational content can be used to address stigma in historically stigmatized groups. Future interventions may build on the present one and feature more robust samples and study designs. The fact that attitudes toward transgender individuals vary highly across cultures provides an opportunity to assess the efficacy of interventions in diverse cultural contexts. We believe that stigma against transgender youth should be explored in more detail—first and foremost due to the demonstrated burden on the mental health of stigmatized children, who deserve the chance to express themselves authentically and thrive in an accepting society.

## Appendix

Multimedia Appendix 1 Overview of course content.

Multimedia Appendix 2 Overview of video content.

Multimedia Appendix 3 Transgender stigma survey.

Multimedia Appendix 4 Adaptation of the Transgender Attitudes and Beliefs Scale (TABS) to the context of parents and caregivers.

Multimedia Appendix 5 Pre- and post-course survey matching based on the unique identifier.

Multimedia Appendix 6 Pre- and post-course survey matching using a propensity score approach.

## Abbreviations

CHERRIES: Checklist for Reporting Results of Internet E-Surveys
TABS: Transgender Attitudes and Beliefs Scale

## Data Availability

The datasets generated during and/or analyzed during this study are available from the corresponding author on reasonable request.
