# Supplementary material for: Effect of a Narrative-Based Online Course Aimed at Reducing Stigma Toward Transgender Children and Adolescents: Longitudinal Observational Study
Source: JMIR Form Res. 2025 Jan 9;9:e59605. doi: 10.2196/59605 (PMC11757976; doi:10.2196/59605)
Supplement: Multimedia Appendix 1 [file formative_v9i1e59605_app1.docx]

# Appendix 1

**Week 1: What is Gender Identity?**

In module one, we will begin to hear some of the real-life experiences of transgender children, their friends and families. We will gain a better understanding of terms like: gender identity, sex assigned at birth and sexual orientation as well as understanding how these characteristics differ from one another. By the end of this module, learners be more familiar with what it means to be transgender and what it means to have a non-binary gender identity.

**Learning Objectives:**

- Empathize with the experience of transgender individuals, including children, their friends and families.
- Define and correctly use relevant terms including: gender identity, sex assigned at birth, sexual orientation, transgender, non-binary gender identity and other commonly conflated terms.
- Explain the difference between gender identity, gender expression and sex assigned at birth.
- Explain the difference between gender identity and sexual orientation as well as when these typically develop.

**Sections:**

What Is the Difference Between Gender Identity and Sex Assigned at Birth?

- Couse Introduction (Video, 3 minutes)
- “Don’t Give Up” (Video, 3 minutes)
- What Is the Difference Between Sex Assigned at Birth and Gender Identity? (Video, 4 minutes)
- “You Amaze Me Every Day” (Video, 2 minutes)
- The Main Point #1 (Practice Quiz, 2 questions)

What Is the Difference Between Gender Identity and Sexual Orientation?

- What Is the Difference Between Gender Identity and Sexual Orientation? (Video, 4 minutes)
- The Main Point #2 (Practice Quiz, 1 question)
- Ben Barres: “It’s No Longer Necessary to Live in the Closet” (Video, 5 minutes)
- Gender and Role Models (Discussion Prompt, 10 minutes)

Week 1 Quiz: Gender Identity (4 questions)

**Week 2: What is the Gender Spectrum?**

In this module, we will explore some of the gender affirming management options available to support the health of transgender children across their lifespan, including the potential benefits of delaying puberty in transgender adolescents. We will examine the medical classification of transgender and take a closer look at how the diagnosis has changed over time as well as the arguments for and against the need for a medical diagnosis.

**Learning Objectives:**

- Consider and use supportive language needed for effective communication with transgender individuals and their families, including use of the correct pronouns and names.
- Summarize the debate around the medical classification of transgender as a psychiatric condition, how the diagnosis has changed over time and the arguments for and against the need for a medical diagnosis.
- Describe the gender-affirming management options for transgender individuals through early childhood, adolescence and adulthood.
- Explain the potential benefits of delaying puberty in transgender adolescents.
- Define and correctly use relevant terms including: gender identity, sex assigned at birth, sexual orientation, transgender, non-binary gender identity and other commonly conflated terms

**Sections:**

What Are the Gender-Affirming Management Options for Transgender Individuals?

- What Are the Gender-Affirming Management Options for Transgender Individuals? (Video, 6 minutes)
- The Main Point #3 (Practice Quiz, 1 question)
- “I Wouldn’t Ask for Any Other Child” (Video, 7 minutes)
- Gender Affirmation (Discussion Prompt, 10 minutes)

How Is Transgender Classified in Medicine?

- How Is Transgender Classified in Medicine? (Video, 4 minutes)
- The Main Point #4 (Practice Quiz, 1 question)
- “It’s Got to Be OK” (Video, 6 minutes)
- Gender Identity and Friendships (Discussion Prompt, 10 minutes)

What Is a Non-Binary Gender Identity?

- What Is a Non-Binary Gender Identity? (Video, 4 minutes)
- The Main Point #5 (Practice Quiz, 1 question)
- “Who Doesn’t Want to Be Heard?” (Video, 6 minutes)
- Your Experiences of Being Supported (Discussion Prompt, 10 minutes)

Week 2 Quiz: The Gender Spectrum (6 questions)

**Week 3: How Do We Create a Gender-Inclusive Society?**

In this module, we will explore some of the concrete steps that healthcare providers, teachers and parents can take to make their clinics, schools and homes more gender inclusive. We will discuss the importance of making basic facilities such as restrooms accessible to all children and we will explore the adverse consequences that can occur when children do not feel safe using public restrooms. We will also discuss the negative health outcomes associated with lack of familial and societal support for the transgender child.

**Learning Objectives:**

- Describe concrete actions healthcare providers can take to make their healthcare setting more gender inclusive.
- Support parents and teachers in creating a gender inclusive school environment.
- Explain the importance of accessibility to basic facilities such as restrooms and the adverse consequences that can occur when children do not feel safe using public restrooms.
- Explain the negative health outcomes associated with lack of familial and societal support for the transgender child.
- Empathize with the experience of transgender individuals, including children, their friends and families.
- Consider and use supportive language needed for effective communication with transgender individuals and their families, including use of the correct pronouns and names.

**Sections:**

How to Create Gender-Inclusive Healthcare Settings

- How to Create Gender-Inclusive Healthcare Settings (Video, 4 minutes)
- The Main Point #6 (Practice Quiz, 1 question)
- “Hold Space for Your Child” (Video, 7 minutes)
- Bathrooms and Bullying (Discussion Prompt, 10 minutes)

How to Create Gender-Inclusive Classroom Settings

- How to Create Gender-Inclusive Classroom Settings (Video, 4 minutes)
- The Main Point #7 (Practice Quiz, 1 question)
- “This Is Real” (Video, 5 minutes)
- Creating Gender Inclusiveness in Your Life (Discussion Prompt, 10 minutes)

Why is Social Support So Important for Transgender Individuals?

- Alice Lyman Miller: “You’ve Only Got One Life” (Video, 6 minutes)
- Why Is Social Support So Important for Transgender Individuals? (Video, 3 minutes)
- The Main Point #8 (Practice Quiz, 1 question)
- “What Is a Girl?” (Video, 7 minutes)
- How Can You Support Transgender Friends and Family? (Discussion Prompt, 10 minutes)

Week 3 Quiz: Creating a Gender Inclusive Society (6 questions)
