# Supplementary figures and images for: Effect of a Narrative-Based Online Course Aimed at Reducing Stigma Toward Transgender Children and Adolescents: Longitudinal Observational Study
Source: JMIR Form Res. 2025 Jan 9;9:e59605. doi: 10.2196/59605 (PMC11757976; doi:10.2196/59605)

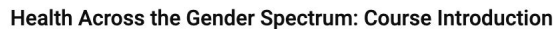

3:12

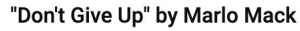

3:16

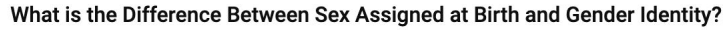

4:37

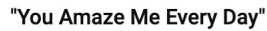

2:42

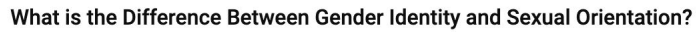

4:14

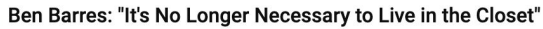

5:02

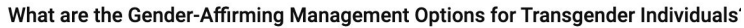

6:16

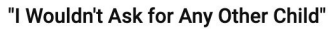

7:42

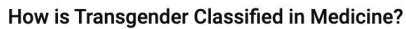

4:05

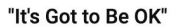

6:25

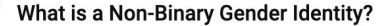

4:30

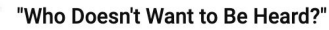

6:36

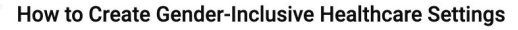

4:27

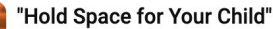

7:17

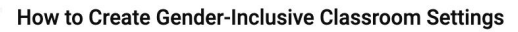

4:36

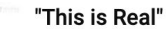

5:2

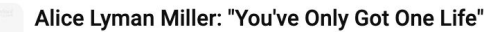

6:1

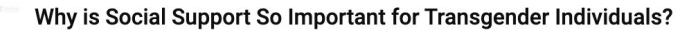

3:4

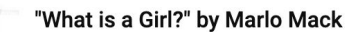

7:0

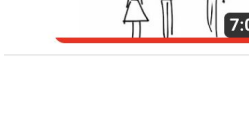

Supplement: Multimedia Appendix 2 [file formative_v9i1e59605_app2.pdf]

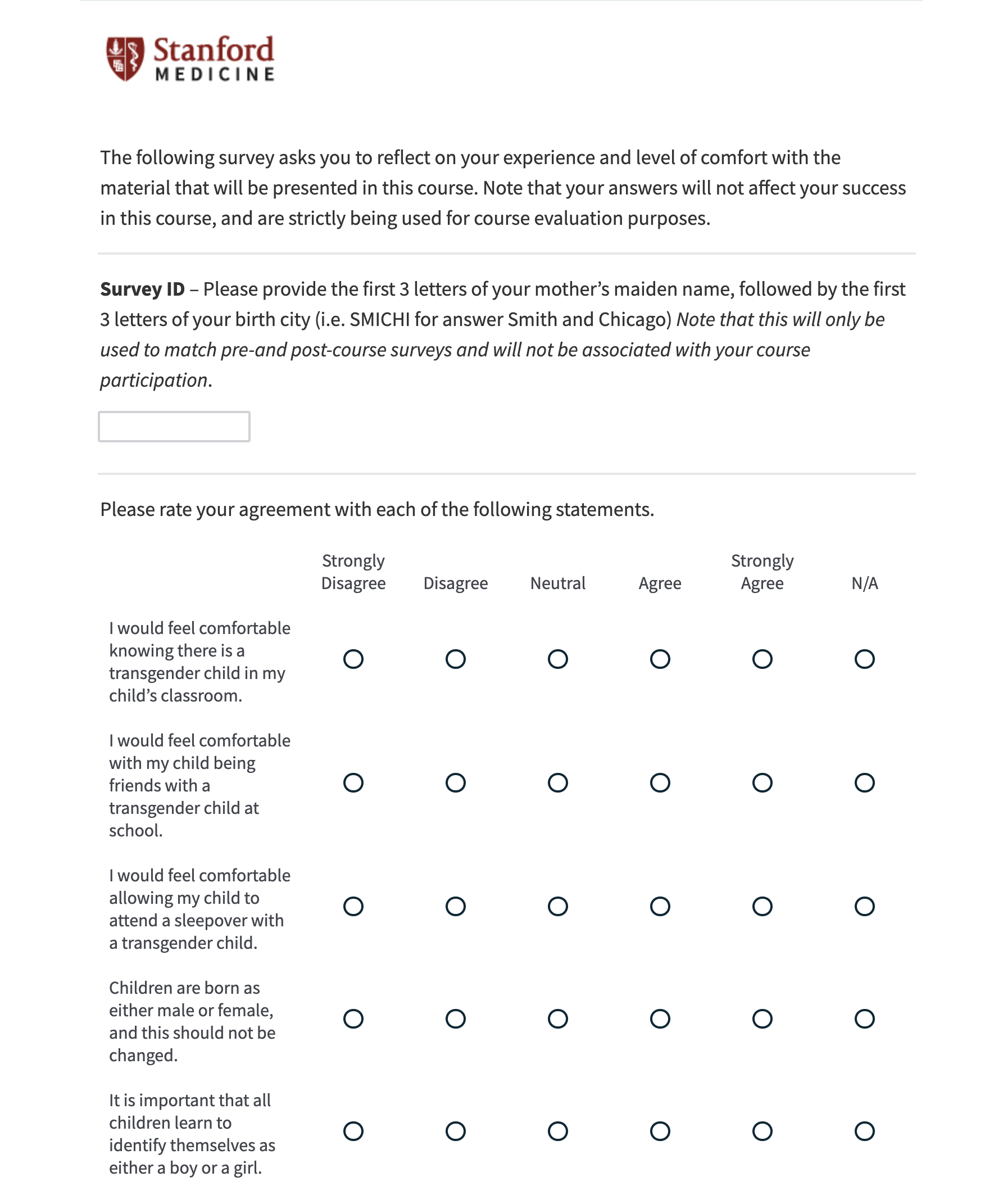

Supplement: Multimedia Appendix 3 [file formative_v9i1e59605_app3.png]
