# Supplementary material for: Effect of a Narrative-Based Online Course Aimed at Reducing Stigma Toward Transgender Children and Adolescents: Longitudinal Observational Study
Source: JMIR Form Res. 2025 Jan 9;9:e59605. doi: 10.2196/59605 (PMC11757976; doi:10.2196/59605)
Supplement: Multimedia Appendix 4 [file formative_v9i1e59605_app4.docx]

# Appendix 4

Adaptation of the Transgender Attitudes and Beliefs Scale (TABS) to the context of parents and caregivers:

1st item in our survey: I would feel comfortable knowing there is a transgender child in my child’s classroom.

Original TABS item (Q3.23., subscale 1): I would feel uncomfortable working closely with a transgender person in my workplace.

2nd item in our survey: I would feel comfortable with my child being friends with a transgender child at school.

Original TABS item (Q3.11., subscale 1): If I knew someone was transgender, I would still be open to forming a friendship with that person.

3rd item in our survey: I would feel comfortable allowing my child to attend a sleepover with a transgender child.

Original TABS item (Q3.21, subscale 1): If my child brought home a transgender friend, I would be comfortable having that person into my home.

4th item in our survey: Children are born as either male or female, and this should not be changed.

Original TABS item (Q3.19., subscale 2): If you are born male, nothing you do will change that.

5th item in our survey: It is important that all children learn to identify themselves as either a boy or a girl.

Original TABS item (Q3.13, subscale 2): All adults should identify as either male or female.
