# Supplementary material for: Effect of a Narrative-Based Online Course Aimed at Reducing Stigma Toward Transgender Children and Adolescents: Longitudinal Observational Study
Source: JMIR Form Res. 2025 Jan 9;9:e59605. doi: 10.2196/59605 (PMC11757976; doi:10.2196/59605)
Supplement: Multimedia Appendix 5 [file formative_v9i1e59605_app5.docx]

# Appendix 5

**Pre- and post-course survey matching based on the unique identifier**

The letter code that respondents’ were asked to generate in a pre-question (Q1) prior to the survey, meant as a unique identifier, could not uniquely identify all of the records. There were cases with

- same ID, same location, different ages,
- same ID, same location, same ages, different answers,
- same IP address, same location, different ID, different answers.

Thus, we merged Q1 using a many-to-many relationship to avoid omitting a valid subject. In total, there were 586 pairs of matched pre- and post-surveys. Subsequently, we checked and removed subjects if any of the following conditions were met:

- EndDate of post-survey before EndDate of pre-survey, indicating a mismatch.
- age of post-survey < age of pre-survey, indicating a mismatch.
- differences in EndDate of pre- and post-survey greater than the age differences, indicating a mismatch.
- sex was not matching in the pre- and post-surveys,
- education was not matching in the pre- and post-surveys,
- race was not matching pre- and post-surveys,
- subregion was not matching in the pre- and post-surveys.

After removing the subjects who did not fulfill these criteria, 447 matched individuals remained. These constituted the fully matched sample. Next, we assigned the values −2,−1,0,1, and 2, respectively, to the answer choices of the Likert scale. Positive scores expressed positive attitudes regarding the questions (“strongly agree” or “agree” for the first three statements, “strongly disagree” or “disagree” for the last two statements), while negative scores referred to negative attitudes. 0 was equivalent to “neutral”.
