# Supplementary material for: Effect of a Narrative-Based Online Course Aimed at Reducing Stigma Toward Transgender Children and Adolescents: Longitudinal Observational Study
Source: JMIR Form Res. 2025 Jan 9;9:e59605. doi: 10.2196/59605 (PMC11757976; doi:10.2196/59605)
Supplement: Multimedia Appendix 6 [file formative_v9i1e59605_app6.docx]

# Appendix 6

**Pre- and post-course survey matching using a propensity score approach**

In the time period between 2017-2023, 1887 participants answered the pre-course survey and 1575 subjects the post-course survey. This includes submissions with incomplete data. Of these submissions, only 447 could be matched. In order to include as many participants as possible, we applied propensity score 1:1 matching for the participants with no missing data for age, sex, race, education, and subregion to match the pre- and post-course surveys. This allows for the comparison between the pre- and post-course surveys for all individuals who could be identified and matched, reducing the potential confounding and model dependence when estimating the course effects. We used the nearest neighbor matching approach which was shown to perform better than others. We matched the “treatment” group (post-course records) with the pre-course records based on the nearest distance on the propensity score, employing sampling with replacement. In total, the matched sample includes 2924 subjects. The demographic distribution of the sample before and after matching with propensity score are shown in Figure 4 in which most of the variables are balanced. Those and the remaining less balanced variables were adjusted by including them as covariates in the regression models.


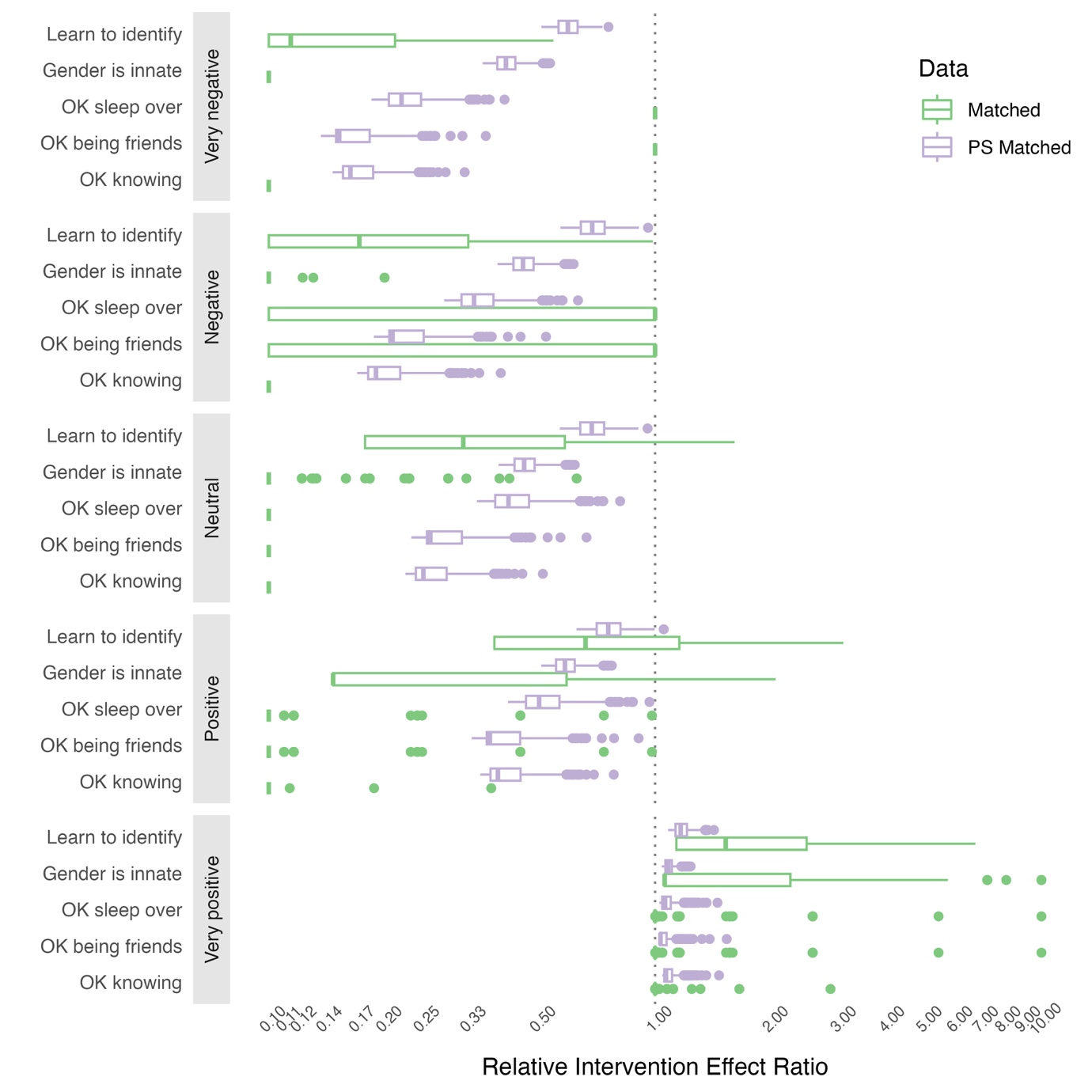


Figure 4: Estimated relative effect ratio of the intervention for each question. The colors represent the model using fully matched data (small sample size, green) versus propensity score (PS) matched data (larger sample size, purple). The dots represent the estimate conditional on the individual effect. We truncated the ratio at an equivalent of 10 times relative difference for visualization purposes, since the predicted probability can be very small.
